# Calibration and Validation of the CSM-CROPGRO-Peanut Model Under Mulched Drip Irrigation Conditions in Xinjiang

**DOI:** 10.3390/plants14040614

**Published:** 2025-02-18

**Authors:** Junwei Chen, Qiang Li, Xiaopei Zhang, Jianshu Dong, Xianfei Hou, Haocui Miao, Haiming Li, Yuchao Zhang, Xiaojun Shen, Zhuanyun Si, Zhijie Shan

**Affiliations:** 1 College of Water Conservancy Engineering, Tianjin Agricultural University-China Agricultural University Joint Smart Water Conservancy Research Center, Tianjin Agricultural University, Tianjin 300392, China; chenjunwei202204@163.com (J.C.); xiaopeihb@163.com (X.Z.); 15228177307@163.com (H.L.); 13820627850@163.com (Y.Z.); shenxiaojun@tjau.edu.cn (X.S.); 2 Institute of Western Agriculture, The Chinese Academy of Agricultural Sciences, Changji 831100, China; 3 Institute of Economic Crops, Xinjiang Academy of Agricultural Sciences, Urumqi 830091, China; lq19820302@126.com (Q.L.); hou544805196@163.com (X.H.); mc09876@163.com (H.M.); 4 College of Water Conservancy & Architectural Engineering, Shihezi University, Shihezi 832000, China; dongjianshu_1010@163.com; 5 Institute of Farmland Irrigation, Chinese Academy of Agricultural Sciences, Xinxiang 453002, China; 6 State Key Laboratory of Simulation and Regulation of Water Cycle in River Basin, China Institute of Water Resources and Hydropower Research, Beijing 100048, China

**Keywords:** DSSAT model, Arachis hypogaea, peanut, water and nitrogen regulation, parameter estimation, model validation

## Abstract

In order to explore the applicability of the peanut growth simulation model CSM-CROPGRO-Peanut under conditions of mulched drip irrigation in Xinjiang, and to determine the optimal scenario for parameter estimation and model validation, field experiments were conducted in 2022 and 2023 on the water and nitrogen regulation of peanut. Based on the water requirements during the stages of peanut growth, three irrigation levels (low, medium, and high) and two nitrogen application levels (100% N and 50% N) were set, resulting in six treatments. An additional control treatment (CK) with a medium irrigation level and no nitrogen application was also included. In this study, four different parameter estimation and validation protocols were designed, and different parameter estimation results were obtained using the DSSAT-GLUE parameter estimation module. The results showed that the FL-SH (time between first flower and first pod), FL-SD (time between first flower and first seed), SIZLF (time between first flower and first seed), XFRT (maximum size of full leaf), and WTPSD (maximum weight per seed) parameters exhibited strong variability, with coefficients of variation of 24.33%, 22.9%, 19.78%, 14.47%, and 23.82%, respectively, and were significantly affected by environment–management interactions. Other parameters showed weaker variability, with coefficients of variation that were all less than 10%. The model outputs varied significantly among different parameter estimation protocols. Scenario 3, which used data from the adequate irrigation and adequate fertilization treatment (W3N2) environment across both years for parameter estimation and data from other treatments for validation, showed the highest model calibration and validation accuracy. The average absolute relative error (ARE) and normalized root mean square error (nRMSE) for model calibration and validation were the lowest at 9.1% and 10.1%, respectively. The CSM-CROPGRO-Peanut model effectively simulated peanut growth and development as well as soil moisture dynamics under mulched drip irrigation conditions in Xinjiang, with the highest simulation accuracy observed under full irrigation conditions. The findings provide a basis for using the CSM-CROPGRO-Peanut model to develop suitable irrigation and nitrogen application regimes for peanuts under mulched drip irrigation in Xinjiang.

## 1. Introduction

Peanut (*Arachis hypogaea* L.), as a crucial economic and oilseed crop in China, holds a significant position in the national economy and is of great importance for its supply of edible oils [1]. The Xinjiang region, characterized by its unique climatic conditions—dry with minimal rainfall, abundant sunshine, and rich light and heat resources—coupled with gray desert soil as the primary soil type, provides a superior natural environment for peanut cultivation. The limited rainfall in this area effectively inhibits the growth of *Aspergillus flavus*, thereby ensuring the high quality of peanut oil, and the peanut crop exhibits strong drought tolerance, which is conducive to achieving a high quality and yield [2]. The peanut growing season in the Xinjiang region spans from April to September, with an annual average precipitation ranging between 100 and 200 mm. Precipitation is primarily concentrated between June and August, with monthly rainfall typically ranging from 10 to 30 mm. During July and August, daytime temperatures generally range from 30 °C to 35 °C, occasionally exceeding 35 °C, while nighttime temperatures typically range between 15 °C and 20 °C. In May and September, temperatures may drop to 5 °C to 10 °C. Due to the limited precipitation, peanut growth is heavily dependent on irrigation.

In recent years, numerous scholars have conducted extensive research on modes of irrigation and nitrogen application for peanuts under film drip irrigation in Xinjiang [3,4,5]. However, traditional field experiment methods are time-consuming, labor-intensive, and costly, and their results have limited general applicability. With the development of information technology and mathematical models, crop growth models are now widely used in agricultural research. Among them, the Decision Support System for Agrotechnology Transfer (DSSAT) is one of the most widely used crop models globally, capable of accurately simulating the daily growth and development processes of crops and responding to various factors such as crop genetic characteristics, field management practices, and environmental conditions [6,7,8,9,10].

Numerous studies have successfully been conducted based on the DSSAT model to optimize the water and nitrogen regulation schedules for different crops. For instance, Si et al. [11] used the DSSAT-CERES-Wheat model to develop optimized irrigation and nitrogen application strategies for drip-irrigated winter wheat in North China. Du et al. [12] determined the optimal irrigation quota for mulched-drip-irrigated cotton in Southern Xinjiang using the DSSAT model. Additionally, Sezen et al. [13] effectively simulated the impact of irrigation levels and frequencies on peanut growth, development, and yield in Turkey in the Eastern Mediterranean region by adjusting and evaluating the CSM-CROPGRO-Peanut model, achieving efficient utilization of irrigation water.

However, model calibration and validation are crucial steps to ensuring the simulation accuracy and reliability of crop models. Different model parameter estimation scenarios yield different parameter values and simulation results [14,15,16]. In terms of model parameter calibration, the traditional trial-and-error method, while simple and straightforward, is subjective and significantly less efficient when dealing with a large number of parameters [17]. Therefore, researchers have developed various systematic algorithms, such as the simplex method [18], the simulated annealing method [19], genetic algorithms [20,21], Bayesian methods [22], and the particle swarm optimization method [23], to optimize model parameter estimation. Among them, the generalized likelihood uncertainty estimation (GLUE) method, a type of Bayesian method, can effectively estimate model parameters by combining prior information with experimental data [24] and has been integrated into DSSAT v4.8 software as a universal method for estimating the cultivation parameters of different crops [25,26].

This study intended to utilize experimental data on the water and nitrogen regulation of peanuts in Xinjiang over two consecutive years (2022 and 2023) to run the CSM-CROPGRO-Peanut model and estimate model genotype parameters using the GLUE method. By comparing and analyzing different model parameter estimation and validation scenarios, the aim was to assess the reliability of the CSM-CROPGRO-Peanut model in simulating peanut growth, development, and yield formation under mulched drip irrigation in Xinjiang and to select the best simulation scenario. The results of this study will provide a scientific basis for using the model to develop suitable irrigation and nitrogen application regimes for peanuts under mulched drip irrigation in Xinjiang and support the widespread application of this model in simulating agricultural ecosystems in arid and semi-arid regions of China.

## 2. Materials and Methods

### 2.1. Experimental Site

**Figure 1:**
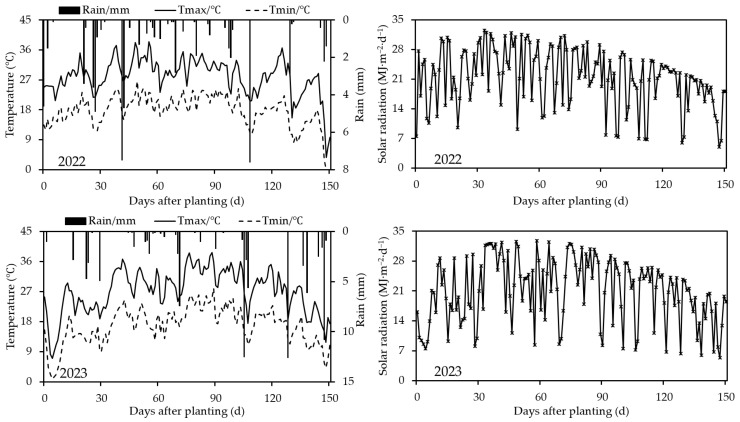
Meteorological data over whole peanut growth period in 2022 and 2023.

Field experiments were conducted from 2022 to 2023 at the Anningqu Comprehensive Experimental Station of Xinjiang Academy of Agricultural Sciences, located in Urumqi, Xinjiang Uyghur Autonomous Region (43°58′ N, 87°30′ E, altitude 590 m). The study area is situated in the middle of the northern foot of the Tianshan Mountains, in an alluvial plain in the northern outskirts of Urumqi, and is characterized by a gentle terrain. It is a typical region of the Tianshan Northern Slope Economic Belt. During the growing season in 2022, the maximum and minimum temperatures ranged from 3.7 to 38.5 °C and from −1.4 to 26.6 °C, respectively, with a total rainfall of 63.1 mm. In 2023, the maximum and minimum temperatures during the growing season were 7.1 to 38.7 °C and 0.9 to 28.0 °C, respectively, with a total rainfall of 95.8 mm, as depicted in Figure 1. The region follows a one-crop-per-year system, exhibiting a state of “insufficient for two crops, surplus for one”. The soil at the experimental site is classified as grey desert soil, with a pH value ranging from 7.8 to 8.0 in the 0–60 cm layer before sowing, an average bulk density of 1.41 g/cm^3^, and a groundwater table depth of 7.5 m. The basic physicochemical parameters of the soil are presented in Table 1.

### 2.2. Experimental Design

**Figure 2:**
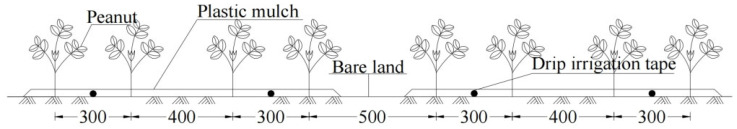
Layout of drip irrigation in peanut field under film mulching (mm).

The test crop was peanut (variety “Huayu 9610”), planted in a wide–narrow row configuration with 1 film covering 2 strips and 4 rows (Figure 2). The film width was 1.25 m, with 4 rows of peanuts planted within each film. The row spacings for the peanuts were 30 + 40 + 30 cm, and the hole spacing was 15 cm. The spacing between peanut rows across two films was 60 cm. The total width for 4 rows of peanuts under a single film was 1.5 m, with a planting density of approximately 1.67 × 10^5^ holes/ha. The sowing dates for peanuts in 2022 and 2023 were May 7 and May 1, respectively. Before sowing, a base compound fertilizer (N-P_2_O_5_-K_2_O = 15-15-15) was applied at a rate of 300 kg/ha. During the growth period, nitrogen fertilizer (urea (CO(NH_2_)_2_, with nitrogen content ≥ 46%)) was applied using a Venturi fertilizer applicator. No additional micronutrients were applied to peanuts during the growth period. Irrigation was carried out using drip irrigation under the film, with water volumes for each treatment measured using flow meters. The irrigation water source was groundwater.

Based on the growth habits of peanuts, the growth period was divided into four stages: seedling, flowering and pegging, pod-setting, and pod-filling. The field experiment was a split-plot design with water and nitrogen as the two factors. The main plot factor was nitrogen application, with two levels: conventional nitrogen application during the growth period (1) and half the conventional nitrogen application during the growth period (0.5) (denoted as N2 and N1, respectively). The subplot factor was irrigation, with three levels set as low, medium, and high water (denoted as W1, W2, and W3, respectively). A complete combination of the two factors resulted in 6 treatments. An additional control treatment (CK) with a medium irrigation level and no nitrogen application was also included, resulting in a total of 7 treatments (Table 2). Each treatment was replicated 3 times. According to local peanut production practices, no irrigation was applied during the seedling stage. To ensure smooth emergence, the peanuts were irrigated with 45 mm of water after sowing to facilitate seedling emergence.

### 2.3. Measurement Items and Methods

#### 2.3.1. Determination of Soil Water Content

The soil water content was determined at a depth of 0–60 cm, in layers of 10 cm each, before sowing, before and after irrigation, and after harvest using the soil drying method. The sampling point was selected directly below the drip irrigation line for each treatment. The method described by Shen et al. [27] was used to calculate the average soil water content of the profile, which represented the average soil water content in the peanut field.

#### 2.3.2. Measurement of Growth Indicators

From the time of peanut sowing, three representative peanut plants from each treatment were selected for measurement at each growth stage, with each treatment replicated three times. The leaf area index (LAI) was determined and calculated using the specific leaf weight method [28]. The aboveground biomass of peanut plants was measured separately for stems and leaves. They were first killed at 105 °C for 30 min and then dried at 75 °C until a constant weight was reached, and the dry weight was measured using an electronic balance with an uncertainty of ± 0.01 g.

#### 2.3.3. Measurement of Pod Yield

After peanut harvest, three representative 6.67 m^2^ plots were selected from each treatment. The pods were picked, placed into mesh bags, air-dried naturally, and weighed. The yield was then converted to pod yield per hectare.

### 2.4. Model Input Data

#### 2.4.1. Meteorological Data

The meteorological data required by the CSM-CROPGRO-Peanut model were obtained from the automatic meteorological monitoring station (HOBO U30 Weather Station, HOBOware 3.7.25 Software, Onset Computer Corporation, Bourne, MA, USA) at the experimental site. The data mainly included daily maximum and minimum temperatures, solar radiation, and rainfall. The Weatherman program built into the model was used to import the data and create WTH meteorological files. The meteorological data for 2022 and 2023 are shown in Figure 1.

#### 2.4.2. Soil Data

Before the start of the field experiment, soil samples were taken at different depths using a soil auger to measure the physical properties and initial conditions of the soil at a depth of 0–60 cm. The SBuild module in the model was used to create the SOL soil file required by the CSM-CROPGRO-Peanut model. The physicochemical properties of the soil in the experimental area are shown in Table 1.

#### 2.4.3. Field Management Data

The field management data mainly included sowing date, planting density and depth, irrigation date and amount, fertilization date and amount, etc. The field management data used in this study were derived from the 2022 and 2023 field experiments on peanut cultivation under film-covered drip irrigation in Xinjiang.

### 2.5. Description, Calibration, and Validation of the CSM-CROPGRO-Peanut Model

The CSM-CROPGRO-Peanut model is a component of the DSSAT software application. It is embedded in the DSSAT-CSM (crop system model) platform and can invoke the generic modules of weather, soil, soil–plant atmosphere (SPAM), and management to simulate crop growth, yield and carbohydrate balance [29]. The main function of the weather module is to read or generate daily weather data. The soil module integrates information from four sub-modules—soil water, soil temperature, soil carbon and nitrogen, and soil dynamics—and calculates and updates data for each soil layer on a daily basis [29]. The SPAM module brings together soil, plant, and atmospheric inputs and calculates root water uptake, potential evapotranspiration (ET), actual soil evapotranspiration, and plant transpiration. ET can be calculated using the Priestley Taylor method [30] or the Penman-FAO method. The default Priestley and Taylor methods were used in our study, requiring only daily solar radiation and temperature. Actual soil evapotranspiration and plant transpiration were calculated by ET, and detailed information on these aspects can be found in Jones et al. [29]. The SPAM module determines when to perform field operations (planting, harvesting, applying inorganic fertilizers, irrigating, and applying crop residues and organic materials) by invoking the sub-modules. The data required to run the model typically consist of four types: weather data, soil data, crop genetic coefficients, and management information.

The DSSAT-GLUE parameter calibration tool was used in this study to estimate the genetic parameters of the peanut variety “Huayu 9610”. The parameters included CSDL, PPSEN, EM-FL, FL-SH, FL-SD, SD-PM, FL-LF, LFMAX, SLAVR, SIZLF, XFRT, WTPSD, SFDUR, SDPDV, PODUR, THRSH, SDPRO, and SDLIP (Table 3). The DSSAT-GLUE tool is based on the generalized likelihood uncertainty estimation (GLUE) method [6,31]. The GLUE method evaluates the credibility of parameters by comparing simulated values with observed data, using likelihood values rather than simply accepting or rejecting potential parameters. This approach is particularly effective for parameter estimation under conditions of significant model and observational uncertainty, thus enhancing the scientific rigor of the estimation [14].

In the GLUE framework, the likelihood function serves as the criterion for evaluating genetic parameters. The program compares the simulated values of each parameter set with the corresponding observed values and calculates their likelihood values. The higher the likelihood value, the better the match between the simulated and observed values. Subsequently, the total likelihood of all related simulations and observations is computed, and Bayesian inference is used to calculate the posterior distribution of the parameter sets [32]. To ensure the accuracy of the parameter estimation and the reliability of the posterior distribution, each GLUE run must involve at least 3000 iterations [33].

The phenological period (including flowering and maturation), final aboveground biomass, pod yield, and maximum peanut leaf area index were taken as the output variables of the model, and the parameters were estimated and verified by comparing the simulated values of the model with the observed values in the field. During the calibration process, initial default values required for the program were set, and 10,000 random searches were conducted using the GLUE tool to obtain a more reliable set of parameter combinations. Considering the influence of temporal and environment–management interactions on field experiments, this study employed different parameter calibration–validation scenarios (Table 4), and through the comparison of simulation results from these scenarios, the optimal scenario was determined.

### 2.6. Model Error Detection

In this study, the model calibration and validation processes were evaluated based on the absolute relative error (ARE), root mean square error (RMSE), normalized root mean square error (nRMSE), and correlation coefficient (R^2^) between the simulated and observed values. These metrics quantify the relative discrepancies between simulated and observed values and are dimensionless statistics, allowing for comparisons across different variables [34]. Lower values of ARE and nRMSE indicate higher model simulation accuracy.

(1) $$\mathrm{ARE}=\frac{\left|S_{i}-O_{i}\right|}{O_{i}}\text{×}100\%$$

(2) $$\mathrm{RMSE}=\sqrt{\sum_{i=1}^{n}\frac{{\left(S_{i}-O_{i}\right)}^{2}}{n}}$$

(3) $$\mathrm{nRMSE}=\frac{\sqrt{\sum_{i=1}^{n}\frac{{\left(S_{i}-O_{i}\right)}^{2}}{n}}}{\overset{-}{O}}\text{×}100$$

(4) $$R^{2}=\frac{{\left[\sum_{i=1}^{n}\left(O_{i}-\overset{-}{O}\right)\text{×}\left(S_{i}-\overset{-}{S}\right)\right]}^{2}}{\sum_{i=1}^{n}{\left(O_{i}-\overset{-}{O}\right)}^{2}\text{×}\sum_{i=1}^{n}{\left(S_{i}-\overset{-}{S}\right)}^{2}}$$

where S_i_ and O_i_ represent the i-th simulated and observed values, respectively; $\overset{-}{S}$ and $\overset{-}{O}$ are the simulated and observed mean values, respectively; and n is the number of data points. The ARE value reveals the relative deviation between the simulated and observed data. A lower ARE value indicates higher model accuracy and precision. When nRMSE < 10%, the simulation accuracy is excellent; when 10% ≤ nRMSE ≤ 20%, the simulation accuracy is good; when 20% ≤ nRMSE ≤ 30%, the simulation accuracy is moderate; and when nRMSE > 30%, the simulation accuracy is poor [8].

## 3. Results

### 3.1. Comparison of Model Parameters Derived from Different Calibration–Validation Scenarios

The parameter estimates obtained from different model parameter estimation scenarios are presented in Table 5.

A comparison of the results across scenarios reveals significant variability in the following parameters: FL-SH (ranging from 5.549 to 9.344), FL-SD (ranging from 13.87 to 23.86), SIZLF (ranging from 13.2 to 19.46), XFRT (ranging from 0.632 to 0.857), and WTPSD (ranging from 0.708 to 1.162). Their coefficients of variation are 24.33%, 22.9%, 19.78%, 14.47%, and 23.82%, respectively, all exceeding 10%. This indicates substantial changes in the values of FL-SH, FL-SD, SIZLF, XFRT, and WTPSD with different model parameter estimation scenarios. Different parameter estimation scenarios essentially represent different peanut growth contexts (such as different irrigation and nitrogen application treatments and different years). Therefore, the estimates of FL-SH, FL-SD, SIZLF, XFRT, and WTPSD are highly dependent on the specific crop growth context, suggesting that environment–management interactions significantly influence the estimated values of these five parameters. The coefficients of variation for other parameters are all below 10%. Specifically, the standard deviations of CSDL, PPSEN, FL-LF, PODUR, THRSH, SDPRO, and SDLIP are all zero, indicating that these parameters are rarely affected by environment–management interactions. The values of these other parameters are relatively consistent across different crop growth contexts, suggesting that the model’s estimates of various parameters for peanuts with subsurface drip irrigation are reliable.

### 3.2. Model Calibration and Validation Results Across Different Scenarios

In this study, we compared and analyzed the differences between simulated and measured values for the flowering stage, maturity stage, pod yield, aboveground biomass, and maximum leaf area index corresponding to four model calibration and validation scenarios. Based on this, we judged the simulation errors of each scenario and selected the optimal model calibration and validation scenario. The results are shown in Table 6. As can be seen from the table, the ARE and nRMSE of the model calibration results for Scenario 3 are 5.8% and 6.2%, respectively, while the ARE and nRMSE of the model validation results are 12.3% and 13.9%, respectively. The average ARE and nRMSE for the calibration and validation of Scenario 3 are 9.1% and 10.1%, respectively, which are the lowest and most stable compared to other scenarios. Therefore, Scenario 3 has the highest accuracy in simulating the growth, development, and yield of peanuts with subsurface drip irrigation in Xinjiang. The specific comparison results for Scenario 3 are shown in Table 7. The results of the calibration and validation of the CSM-CROPGRO-Peanut model under different scenario conditions (Tables S1–S4) can be found in the Supplementary Materials.

Under the model calibration conditions of Scenario 3, the average ARE values for the peanut flowering stage, maturity stage, pod yield, and aboveground biomass are 1.2%, 3.0%, 4.4%, and 6.6%, respectively, all less than 10%, indicating the high accuracy of the simulation results. However, the average ARE value for the maximum leaf area index is 14.0%, greater than 10%, indicating the lower accuracy of these simulation results. Under model validation conditions, the average ARE values for the peanut flowering stage, maturity stage, pod yield, aboveground biomass, and maximum leaf area index are 1.7%, 3.8%, 18.0%, 18.5%, and 19.7%, respectively, all higher than the average ARE values under model calibration conditions. This suggests that the simulation accuracy under model validation conditions is slightly lower than that under calibration conditions. Among them, Scenario 4 has the largest error in the model calibration results, which is because the treatments used for model calibration were all low-water treatments. This further demonstrates that the CSM-CROPGRO-Peanut model is less accurate in simulating peanut growth under water stress conditions. Overall, Scenario 3 has a relatively high simulation accuracy, and the CSM-CROPGRO-Peanut model performs well under subsurface drip irrigation conditions in Xinjiang, making it the most ideal model calibration and validation scenario.

### 3.3. Simulation Results of Dynamic Variables Under Scenario 3

**Figure 3:**
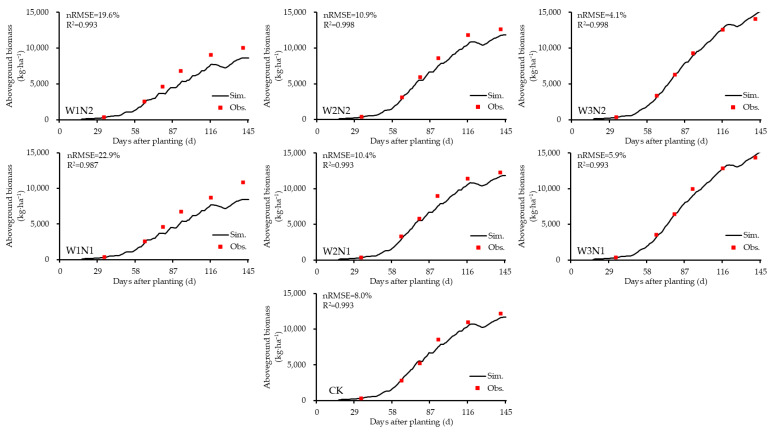
Dynamic simulation of peanut aboveground biomass under mulched drip irrigation in Xinjiang in 2023. Note: Sim. and Obs. represent the simulated and observed values, the same below.

**Figure 4:**
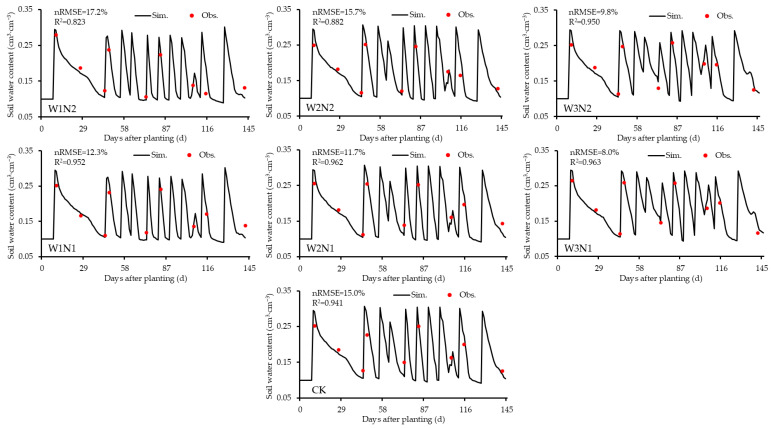
Simulation of peanut soil moisture dynamics under mulched drip irrigation in Xinjiang in 2023.

To further explore the differences in simulation accuracy across different model calibration–validation scenarios, this study compared the simulation results of some important dynamic variables across scenarios. Due to the high simulation accuracy of Scenario 3, it was taken as an example. The genetic parameters obtained from model calibration under this scenario and the relevant experimental data from 2023 were used to run the CSM-CROPGRO-Peanut model. A comparative analysis was conducted on the dynamic changes in peanut aboveground biomass and soil moisture (Figure 3 and Figure 4). Since peanut roots are mostly distributed in the surface soil layer (0–30 cm) [35], for brevity, this study selected the soil moisture in the middle 10–20 cm soil layer for dynamic analysis.

Under the same nitrogen application rate, the normalized root mean square error (nRMSE) for simulating peanut aboveground biomass across treatments was as follows: W3N2 < W2N2 < W1N2 < 20%, W3N1 < W2N1 < W1N1 < 25%, and CK < 10%, with all R^2^ values exceeding 0.9. For soil moisture, the nRMSE followed a similar pattern: W3N2 < W2N2 < W1N2 < 20%, W3N1 < W2N1 < W1N1 < 20%, and CK < 20%, with all R^2^ values exceeding 0.8. Among these treatments, the W3 treatment exhibited the lowest nRMSE and the highest R^2^ values for both aboveground biomass and soil moisture compared to W1 and W2. Specifically, in the W3N2 and W3N1 treatments, the nRMSE for aboveground biomass was 4.1% and 5.9%, respectively, with corresponding R^2^ values of 0.998 and 0.993. For soil moisture, the nRMSE was 9.8% and 8.0%, respectively, with R^2^ values of 0.950 and 0.963. These results demonstrate that under adequate irrigation conditions, the dynamic simulation of peanut aboveground biomass and soil moisture was highly accurate, with the simulation accuracy for aboveground biomass exceeding that for soil moisture. The overall simulation trend in soil moisture was consistent with the observations, and most measured values closely matched the simulated values (Figure 4). Therefore, the CSM-CROPGRO-Peanut model is effective in simulating dynamic changes in peanut aboveground biomass and soil moisture under adequate water supply. At the same irrigation level, the nRMSE for simulating aboveground biomass across treatments was observed to follow this pattern: W1N2 < W1N1, CK < W2N1 < W2N2, and W3N2 < W3N1. For soil moisture, the nRMSE followed a different pattern: W1N1 < W1N2, W2N1 < CK < W2N2, and W3N1 < W3N2. This indicates that increasing the nitrogen application rate reduces the model’s accuracy in simulating soil moisture.

In summary, except for the aboveground biomass in the low-water, low-nitrogen treatment (W1N1), the nRMSE for simulating aboveground biomass and soil moisture across all other treatments using the CSM-CROPGRO-Peanut model was less than 20%, with all R^2^ values exceeding 0.8. These findings confirm that the model meets the requirements for accurately simulating peanut growth and development under mulched drip irrigation conditions in Xinjiang.

## 4. Discussion

### 4.1. Impact of Environment-Management Interactions on Genetic Parameter Estimation in Peanut and Enhancement of Model Accuracy

During model operation, the selection of crop variety parameters directly influences the effectiveness of model simulations. Therefore, the determination of crop variety parameters plays a crucial role in the accuracy of crop model simulations [36]. Crop variety parameters are generally not directly accessible and are often obtained through various parameter estimation methods. Among these methods, the GLUE method has been integrated into the DSSAT model as a built-in parameter estimation tool, allowing its direct use [37]. In this study, DSSAT-GLUE was employed to estimate the genetic parameters of peanuts under film-covered drip irrigation conditions in Xinjiang. A total of four different model calibration–validation scenarios (Table 4) were adopted. The results from the different calibration–validation scenarios underscore the importance of environment–management interactions in model parameterization, especially for parameters such as FL-SH, FL-SD, SIZLF, XFRT, and WTPSD, which exhibited substantial variability across different scenarios. These parameters demonstrated coefficients of variation (CV) exceeding 10% [14], which is indicative of their sensitivity to varying environmental conditions, such as irrigation and nitrogen application regimes. This finding is consistent with previous studies emphasizing the dynamic nature of crop growth under diverse agronomic management practices [14,37]. The significant variation in these parameters suggests that their accurate estimation requires a robust calibration process tailored to specific environmental contexts. It is evident that under varying field management practices, particularly irrigation and nitrogen treatments, the model’s predictive accuracy is enhanced by re-estimating these parameters to capture the complexity of environment–management interactions. The DSSA-GLUE parameter estimation module has good convergence and high reliability when estimating parameters with a coefficient of variation of less than 10%, indicating that some parameters (such as CSDL, PPSEN, FL-LF, PODUR, THRSH, SDPRO, and SDLIP) are relatively unaffected by environmental variability. This stability highlights the robustness of the model in predicting these parameters across different growth conditions. This is consistent with the findings of Fan et al. [38], who observed that certain genetic and physiological parameters remain relatively constant under varying environmental conditions. Thus, these parameters can be reliably used in the model without the need for frequent re-estimation, ensuring stable predictions for long-term simulations or extrapolations across similar environments.

### 4.2. Optimizing Peanut Growth Simulation Under Mulched Drip Irrigation in Xinjiang

Further analysis of the model calibration and validation scenarios revealed that Scenario 3 exhibited the highest accuracy in simulating peanut growth dynamics. The fact that it has the lowest ARE and nRMSE in both calibration and validation (9.1% and 10.1%, respectively) supports the conclusion that this scenario is the most suitable for simulating peanut growth under subsurface drip irrigation in Xinjiang. It is worth noting that the model performed best under fully irrigated conditions, which is reflected in the lower simulation error in the calibration phase. However, a marked decrease in accuracy was observed when the model was validated using water-stressed treatments, particularly in the simulation of aboveground biomass and maximum leaf area index. This indicates that using treatments under fully irrigated conditions for model calibration can achieve a relatively high simulation accuracy, but some errors will be encountered when simulating peanut growth under water stress conditions. This aligns with the findings of Yao et al. [14] and Boote et al. [39], who observed that crop simulation models often struggle with water stress conditions due to their complex effects on plant growth dynamics. The model’s performance, as indicated by the nRMSE values for aboveground biomass and soil moisture, was generally excellent for most treatments under Scenario 3. The fact that the lowest nRMSE and highest R^2^ values for aboveground biomass and soil moisture were observed under W3 treatments confirms the model’s robustness under optimal irrigation conditions. This high accuracy can be attributed to the model’s ability to simulate the dynamic changes in peanut growth under subsurface drip irrigation, a crucial method for improving water use efficiency in semi-arid regions such as Xinjiang [12,40]. The results show that the CSM-CROPGRO-Peanut model can effectively simulate crop growth and water dynamics under controlled irrigation systems, which is of great significance for optimizing water resource utilization and improving agricultural sustainability in water-scarce areas. However, while the model performs well under normal irrigation conditions, its low accuracy under water stress scenarios, especially for aboveground biomass and soil moisture, suggests limitations in its current form [41,42]. This highlights the need to further improve the model’s representation of the effects of water stress on crop growth, especially under variable nitrogen supply conditions. Under the same irrigation level, the accuracy of soil moisture simulations decreased with the increase in nitrogen application. This effect may be due to the complex interaction between nitrogen application and the soil water holding capacity, which makes it difficult for the model to accurately capture the water dynamics after nitrogen application [43]. Therefore, future research could focus on improving the model’s water stress simulation capabilities by incorporating more-dynamic soil–plant–atmosphere interactions and better accounting for nitrogen management strategies.

In conclusion, the CSM-CROPGRO-Peanut model is an effective tool for simulating peanut growth under drip irrigation in Xinjiang under Scenario 3, which is helpful for understanding the dynamic interaction between irrigation, nitrogen application, and crop performance. The model’s accuracy in simulating aboveground biomass and soil water dynamics further supports its potential for application in precision agriculture, where accurate predictions are critical for optimizing water and nutrient management strategies. However, improvements are needed in simulating growth under water constraints, especially in predicting biomass and soil moisture under water stress.

## 5. Conclusions

This study calibrated and validated the CSM-CROPGRO-Peanut model through four distinct scenarios utilizing field experiment data on peanut cultivation under film-covered drip irrigation in Xinjiang from 2022 and 2023. Based on the research findings, the following key conclusions can be drawn:

(1) The genetic parameters FL-SH, FL-SD, SIZLF, XFRT, and WTPSD exhibited significant variability and were significantly affected by environment–management interactions. Therefore, when applying the model to simulation scenarios with substantial variations in field irrigation and nitrogen management conditions, these five parameters should be re-estimated. Otherwise, they may introduce considerable errors in model simulations.
(2) When employing parameter estimation Scenario 3 (using data from the well-watered and well-fertilized treatment W3N2 samples in both years for parameter estimation and data from other treatments for validation), the average ARE and nRMSE for model calibration and validation were 9.1% and 10.1%, respectively, representing the lowest average values for both ARE and nRMSE for both calibration and validation. Scenario 3 was simulated with high accuracy, while the other scenarios were simulated with a relatively low accuracy.
(3) The CSM-CROPGRO-Peanut model effectively simulates the growth and development processes of peanuts and the dynamic changes in soil moisture under film-covered drip irrigation conditions in Xinjiang. The model achieves the highest simulation accuracy under full irrigation conditions, providing a theoretical foundation for predicting the growth, development, and yield of peanuts under varying levels of film-covered drip irrigation and nitrogen application in Xinjiang.

## Figures and Tables

**Table 1:** Soil physical and chemical properties.

Depth (cm)	Clay/%	Silt/%	Sand/%	Field Capacity (cm^3^·cm^−3^)	Saturated Water Content (cm^3^·cm^−3^)	Permanent Wilting Point (cm^3^·cm^−3^)	Available P (mg·kg^−1^)	Available K (mg·kg^−1^)	Alkaline Hydrolysis N (mg·kg^−1^)	Organic Matter (g·kg^−1^)	Bulk Density (g·cm^−3^)
0–20	11.85	43.74	44.42	0.270	0.405	0.11	23.77	199.06	42.98	14.86	1.35
20–40	13.05	45.71	41.25	0.286	0.429	0.10	22.17	169.33	32.04	14.28	1.43
40–60	12.38	42.98	44.64	0.288	0.432	0.10	22.86	114.46	43.41	9.73	1.44

**Table 2:** Irrigation and fertilization regimes for peanuts.

Treatment	Irrigation Quota (mm)					Irrigation Cycle (d)				Nitrogen Fertilizer Rate
	Sowing–Emergence Stage	Seedling Stage	Flowering–Pegging Stage	Pod-Setting Stage	Pod-Filling Stage	Seedling Stage	Flowering–Pegging Stage	Pod-Setting Stage	Pod-Filling Stage	
W1N2	45	-	low	low	low	-	10	10	15	1
W2N2	45	-	medium	medium	medium	-	10	10	15	1
W3N2	45	-	high	high	high	-	10	10	15	1
W1N1	45	-	low	low	low	-	10	10	15	0.5
W2N1	45	-	medium	medium	medium	-	10	10	15	0.5
W3N1	45	-	high	high	high	-	10	10	15	0.5
CK	45	-	medium	medium	medium	-	10	10	15	0

Note: The irrigation quotas for 2022 and 2023 are 22.5, 30, 37.5 mm and 30, 37.5, 45 mm for low, medium, and high water, respectively. A nitrogen fertilizer rate of 1 indicates conventional nitrogen application, 0.5 indicates halved nitrogen application, and 0 indicates no nitrogen application.

**Table 3:** Genetic parameters of the CSM-CROPGRO-Peanut model for the variety “Huayu 9610”.

Parameter	Description	Unit
CSDL	Critical short day length below which reproductive development progresses with no day length effect (for short-day plants)	hour
PPSEN	Slope of the relative response of development to photoperiod with time (positive for short-day plants)	l/hour
EM-FL	Time between plant emergence and flower appearance (R1)	photothermal days
FL-SH	Time between first flower and first pod (R3)	photothermal days
FL-SD	Time between first flower and first seed (R5)	photothermal days
SD-PM	Time between first seed (R5) and physiological maturity (R7)	photothermal days
FL-LF	Time between first flower (R1) and end of leaf expansion	photothermal days
LFMAX	Maximum leaf photosynthesis rate at 30 C, 350 vpm CO_2_, and high light	mg CO_2_·m^2^·s^−1^
SLAVR	Specific leaf area of cultivar under standard growth conditions	cm^2^·g^−1^
SIZLF	Maximum size of full leaf (three leaflets)	cm^2^
XFRT	Maximum fraction of daily growth that is partitioned into seed + shell	g
WTPSD	Maximum weight per seed	g
SFDUR	Seed filling duration for pod cohort at standard growth conditions	photothermal days
SDPDV	Average seed per pod under standard growing conditions	grain/pod
PODUR	Time required for cultivar to reach final pod load under optimal conditions	photothermal days
THRSH	The maximum ratio of [seed/(seed + shell)] at maturity	%
SDPRO	Fraction protein in seeds	g(protein)/g(seed)
SDLIP	Fraction oil in seeds	g(oil)/g(seed)

**Table 4:** Different calibration–validation scenarios for the CSM-CROPGRO-Peanut model.

Scenario	Data for Model Calibration	Data for Model Validation
1	All treatments in 2022	All treatments in 2023
2	All treatments in 2023	All treatments in 2022
3	Two years of full water and fertilizer treatment (W3N2)	All other processing
4	Two years of low-water and low-fertilizer treatment (W1N1)	All other processing

**Table 5:** Genetic parameters of peanuts under different model parameter estimation scenarios.

Parameter	Calibration Result				Mean	Standard Deviation	Coefficient of Variation/%
	1	2	3	4			
CSDL	11.84	11.84	11.84	11.84	11.84	0	0
PPSEN	0	0	0	0	0	0	—
EM-FL	15.52	15.04	15.19	15.17	15.23	0.20	1.3
FL-SH	6.499	5.549	6.198	9.344	6.90	1.68	24.33
FL-SD	22.99	23.66	13.87	23.86	21.10	4.83	22.9
SD-PM	63.79	59.1	69.84	60.25	63.25	4.83	7.63
FL-LF	73.0	73.0	73.0	73.0	73.0	0	0
LFMAX	1.496	1.488	1.438	1.492	1.479	0.027	1.84
SLAVR	278.2	284.4	286.2	274	280.7	5.63	2.01
SIZLF	19.46	13.2	18.41	13.7	16.2	3.20	19.78
XFRT	0.719	0.645	0.632	0.857	0.713	0.10	14.47
WTPSD	1.162	0.736	0.886	0.708	0.873	0.21	23.82
SFDUR	38.28	37.88	41.15	45.39	40.68	3.46	8.5
SDPDV	1.824	1.628	1.949	1.655	1.764	0.15	8.55
PODUR	15.0	15.0	15.0	15.0	15.0	0	0
THRSH	74.5	74.5	74.5	74.5	74.5	0	0
SDPRO	0.27	0.27	0.27	0.27	0.27	0	0
SDLIP	0.51	0.51	0.51	0.51	0.51	0	0

Note: Specific meaning of the parameters are shown in Table 3.

**Table 6:** Simulation accuracies of different scenarios of model calibration and validation.

Scenario	Model Calibration		Model Validation		Average ARE/%	Average nRMSE/%
	ARE/%	nRMSE/%	ARE/%	nRMSE/%		
1	6.8	8.2	13.3	14.8	10.0	11.5
2	8.2	9.4	10.0	11.2	9.1	10.3
3	5.8	6.2	12.3	13.9	9.1	10.1
4	14.3	16.8	12.8	15.4	13.5	16.1

**Table 7:** Results of calibration and validation of the CSM-CROPGRO-Peanut model under Scenario 3 conditions.

Process	Year	Treatment	Anthesis Date (d)			Maturity Date (d)			Pod Yield (kg·ha^−1^)			Aboveground Biomass (kg·ha^−1^)			Maximum Leaf Area Index (cm^2^·cm^−2^)		
			Sim.	Obs.	ARE/%	Sim.	Obs.	ARE/%	Sim.	Obs.	ARE/%	Sim.	Obs.	ARE/%	Sim.	Obs.	ARE/%
Model calibration	2022	W3N2	32	32	0	126	132	4.5	5056	5297	4.5	11,208	11,869	5.6	6.47	7.34	11.9
	2023	W3N2	43	42	2.4	146	144	1.4	5784	5544	4.3	15,156	14,078	7.7	7.44	8.88	16.2
		Average			1.2			3.0			4.4			6.6			14.0
Model validation	2022	W1N2	32	31	3.2	122	132	7.6	1689	2749	38.6	4894	8712	43.8	3.18	4.47	28.9
		W2N2	32	31	3.2	124	132	6.1	3275	4523	27.6	8287	10,409	20.4	5.34	6.56	18.6
		W1N1	32	31	3.2	122	132	7.6	1679	2249	25.3	4837	6709	27.9	3.1	3.88	20.1
		W2N1	32	32	0	124	132	6.1	3186	3898	18.3	8129	9257	12.2	5.32	5.78	8.0
		W3N1	32	31	3.2	126	132	4.5	4890	4698	4.1	11,167	10,354	7.9	6.62	6.86	3.5
		CK	32	33	3.0	124	132	6.1	2801	3398	17.6	7649	8255	7.3	4.83	5.63	14.2
	2023	W1N2	43	43	0	141	144	2.1	2974	4495	33.8	8630	12,442	30.6	5.26	7.38	28.7
		W2N2	43	42	2.4	143	144	0.7	5056	5242	3.5	11,860	13,630	13.0	6.71	8.82	23.9
		W1N1	43	43	0	141	144	2.1	2850	4347	34.4	8439	12,352	31.7	5.14	7.33	29.9
		W2N1	43	43	0	143	144	0.7	4879	5128	4.9	11,826	14,289	17.2	6.66	8.58	22.4
		W3N1	43	42	2.4	146	144	1.4	5752	5414	6.2	15,154	14,343	5.7	7.49	9.03	17.1
		CK	43	43	0	143	144	0.7	4717	4618	2.1	11,692	12,183	4.0	6.49	8.19	20.8
		Average			1.7			3.8			18.0			18.5			19.7

Note: Sim. and Obs. represent the simulated and observed values.

## Data Availability

The data that support the findings of this study are available from the corresponding author, [Z.S. (Zhuanyun Si)].

## Supplementary Materials

The following supporting information can be downloaded at: www.mdpi.com/xxx/s1–4, Tables S1–S4: Results of calibration and validation of the CSM-CROPGRO-Peanut model under different scenario conditions.
