# Supplementary material for: Calibration and Validation of the CSM-CROPGRO-Peanut Model Under Mulched Drip Irrigation Conditions in Xinjiang
Source: Plants (Basel). 2025 Feb 18;14(4):614. doi: 10.3390/plants14040614 (PMC11859345; doi:10.3390/plants14040614)
Supplement: Supplementary file 1 [file plants-14-00614-s001.zip › plants-3390147-supplementary.pdf]

Table S1. Results of calibration and validation of the CSM-CROPGRO-Peanut model under Scenario 1 conditions.

**Process**	**Year**	**Treatment**	**Anthesis date (d)**			**Maturity date (d)**			**Pod yield (kg·ha^{-1})**			**Aboveground biomass (kg·ha^{-1})**			**Maximum leaf area index (cm^2·cm^{-2})**		
			**Sim.**	**Obs.**	**ARE/%**	**Sim.**	**Obs.**	**ARE/%**	**Sim.**	**Obs.**	**ARE/%**	**Sim.**	**Obs.**	**ARE/%**	**Sim.**	**Obs.**	**ARE/%**
Model calibration	2022	W1N2	32	31	3.2	132	132	0	2074	2749	24.6	5408	8712	37.9	3.65	4.47	18.3
		W2N2	32	31	0	132	132	0	3793	4523	16.1	8761	10409	15.8	5.95	6.56	9.3
		W3N2	32	32	0	134	132	1.5	5462	5297	3.1	11997	11869	1.1	7.44	7.34	1.4
		W1N1	32	31	3.2	132	132	0	2075	2249	7.7	5331	6709	20.5	3.57	3.88	8.0
		W2N1	32	32	0	132	132	0	3647	3898	6.4	8602	9257	7.1	5.89	5.78	1.9
		W3N1	32	31	3.2	134	132	1.5	5296	4698	12.7	11940	10354	15.3	7.49	6.86	9.2
		CK	32	33	3.0	132	132	0	3379	3398	0.6	8203	8255	0.6	5.44	5.63	3.4
		Average			1.8			0.4			10.2			14.1			7.4
Model validation	2023	W1N2	44	43	2.3	154	144	6.9	3584	4495	20.3	8535	12442	31.4	5.21	7.38	29.4
		W2N2	44	42	4.8	155	144	7.6	5662	5242	8.0	12036	13630	11.7	7.06	8.82	20.0
		W3N2	44	42	4.8	157	144	9.0	7483	5544	35.0	14826	14078	5.3	7.81	8.88	12.0
		W1N1	44	43	2.3	154	144	6.9	3406	4347	21.6	8361	12352	32.3	5.1	7.33	30.4
		W2N1	44	43	2.3	155	144	7.6	5547	5128	8.2	12004	14289	16.0	7.04	8.58	17.9
		W3N1	44	42	4.8	157	144	9.0	7427	5414	37.2	14810	14343	3.3	7.88	9.03	12.7
		CK	44	43	2.3	155	144	7.6	5364	4618	16.2	11841	12183	2.8	6.85	8.19	16.4
		Average			3.4			7.8			20.9			14.7			19.8

Note: Sim. and Obs. represent the simulated and observed values.

Table S2. Results of calibration and validation of the CSM-CROPGRO-Peanut model under Scenario 2 conditions.

**Process**	**Year**	**Treatment**	**Anthesis date (d)**			**Maturity date (d)**			**Pod yield (kg·ha^{-1})**			**Aboveground biomass (kg·ha^{-1})**			**Maximum leaf area index (cm^2·cm^{-2})**		
			**Sim.**	**Obs.**	**ARE/%**	**Sim.**	**Obs.**	**ARE/%**	**Sim.**	**Obs.**	**ARE/%**	**Sim.**	**Obs.**	**ARE/%**	**Sim.**	**Obs.**	**ARE/%**
Model calibration	2023	W1N2	43	43	0	142	144	1.4	3007	4495	33.1	9478	12442	23.8	6.16	7.38	16.5
		W2N2	43	42	0	143	144	0.7	4977	5242	5.1	12686	13630	6.9	7.74	8.82	12.2
		W3N2	43	42	2.4	145	144	0.7	6570	5544	18.5	15747	14078	11.9	8.65	8.88	2.6
		W1N1	43	43	0	142	144	1.4	2973	4347	31.6	9337	12352	24.4	6.02	7.33	17.9
		W2N1	43	43	0	143	144	0.7	4851	5128	5.4	12670	14289	11.3	7.79	8.58	9.2
		W3N1	43	42	2.4	145	144	0.7	6541	5414	20.8	15754	14343	9.8	8.76	9.03	3.0
		CK	43	43	0	143	144	0.7	4759	4618	3.1	12514	12183	2.7	7.62	8.19	7.0
		Average			0.7			0.9			16.8			13.0			9.8
Model validation	2022	W1N2	32	31	3.23	125	132	5.3	1817	2749	33.9	5411	8712	37.9	3.41	4.47	23.7
		W2N2	32	31	3.23	125	132	5.3	3075	4523	32.0	8864	10409	14.8	5.88	6.56	10.4
		W3N2	32	32	0	126	132	4.5	4668	5297	11.9	12012	11869	1.2	7.42	7.34	1.1
		W1N1	32	31	3.23	125	132	5.3	1812	2249	19.4	5361	6709	20.1	3.35	3.88	13.7
		W2N1	32	32	0	125	132	5.3	2993	3898	23.2	8706	9257	6.0	5.79	5.78	0.2
		W3N1	32	31	3.23	126	132	4.5	4574	4698	2.6	11933	10354	15.3	7.44	6.86	8.5
		CK	32	33	3.03	125	132	5.3	2802	3398	17.5	8282	8255	0.3	5.35	5.63	5.0
		Average			2.28			5.1			20.1			13.7			8.9

Note: Sim. and Obs. represent the simulated and observed values.

Table S3. Results of calibration and validation of the CSM-CROPGRO-Peanut model under Scenario 3 conditions.

**Process**	**Year**	**Treatment**	**Anthesis date (d)**			**Maturity date (d)**			**Pod yield (kg·ha^{-1})**			**Aboveground biomass (kg·ha^{-1})**			**Maximum leaf area index (cm^2·cm^{-2})**		
			**Sim.**	**Obs.**	**ARE/%**	**Sim.**	**Obs.**	**ARE/%**	**Sim.**	**Obs.**	**ARE/%**	**Sim.**	**Obs.**	**ARE/%**	**Sim.**	**Obs.**	**ARE/%**
Model calibration	2022	W3N2	32	32	0	126	132	4.5	5056	5297	4.5	11208	11869	5.6	6.47	7.34	11.9
	2023	W3N2	43	42	2.4	146	144	1.4	5784	5544	4.3	15156	14078	7.7	7.44	8.88	16.2
		Average			1.2			3.0			4.4			6.6			14.0
Model validation	2022	W1N2	32	31	3.2	122	132	7.6	1689	2749	38.6	4894	8712	43.8	3.18	4.47	28.9
		W2N2	32	31	3.2	124	132	6.1	3275	4523	27.6	8287	10409	20.4	5.34	6.56	18.6
		W1N1	32	31	3.2	122	132	7.6	1679	2249	25.3	4837	6709	27.9	3.1	3.88	20.1
		W2N1	32	32	0	124	132	6.1	3186	3898	18.3	8129	9257	12.2	5.32	5.78	8.0

		W3N1	32	31	3.2	126	132	4.5	4890	4698	4.1	11167	10354	7.9	6.62	6.86	3.5
		CK	32	33	3.0	124	132	6.1	2801	3398	17.6	7649	8255	7.3	4.83	5.63	14.2
	2023	W1N2	43	43	0	141	144	2.1	2974	4495	33.8	8630	12442	30.6	5.26	7.38	28.7
		W2N2	43	42	2.4	143	144	0.7	5056	5242	3.5	11860	13630	13.0	6.71	8.82	23.9
		W1N1	43	43	0	141	144	2.1	2850	4347	34.4	8439	12352	31.7	5.14	7.33	29.9
		W2N1	43	43	0	143	144	0.7	4879	5128	4.9	11826	14289	17.2	6.66	8.58	22.4
		W3N1	43	42	2.4	146	144	1.4	5752	5414	6.2	15154	14343	5.7	7.49	9.03	17.1
		CK	43	43	0	143	144	0.7	4717	4618	2.1	11692	12183	4.0	6.49	8.19	20.8
		Average			1.7			3.8			18.0			18.5			19.7

Note: Sim. and Obs. represent the simulated and observed values.

Table S4. Results of calibration and validation of the CSM-CROPGRO-Peanut model under Scenario 4 conditions.

**Process**	**Year**	**Treatment**	**Anthesis date (d)**			**Maturity date (d)**			**Pod yield (kg·ha^{-1})**			**Aboveground biomass (kg·ha^{-1})**			**Maximum leaf area index (cm^2·cm^{-2})**		
			**Sim.**	**Obs.**	**ARE/%**	**Sim.**	**Obs.**	**ARE/%**	**Sim.**	**Obs.**	**ARE/%**	**Sim.**	**Obs.**	**ARE/%**	**Sim.**	**Obs.**	**ARE/%**
Model calibration	2022	W1N1	32	31	3.2	126	132	4.5	1695	2249	24.6	5544	6709	17.4	3.32	3.88	14.4
	2023	W1N1	43	43	0	144	144	0	3635	4347	16.4	8888	12352	28.0	4.81	7.33	34.4
		Average			1.6			2.3			20.5			22.7			24.4
Model validation	2022	W1N2	32	31	3.2	126	132	4.5	1714	2749	37.7	5571	8712	36.1	3.37	4.47	24.6
		W2N2	32	31	0	127	132	3.8	3433	4523	24.1	8927	10409	14.2	5.28	6.56	19.5
		W3N2	32	32	0	127	132	3.8	5610	5297	5.9	11576	11869	2.5	6.12	7.34	16.6
		W2N1	32	32	0	127	132	3.8	3330	3898	14.6	8777	9257	5.2	5.22	5.78	9.7
		W3N1	32	31	3.2	127	132	3.8	5492	4698	16.9	11523	10354	11.3	6.13	6.86	10.6
		CK	32	33	3.0	127	132	3.8	3174	3398	6.6	8126	8255	1.6	4.56	5.63	19.0
	2023	W1N2	43	43	0	144	144	0	3776	4495	16.0	9018	12442	27.5	4.86	7.38	34.1
		W2N2	43	42	2.4	148	144	2.8	6247	5242	19.2	11813	13630	13.3	6.27	8.82	28.9
		W3N2	43	42	2.4	151	144	4.9	8430	5544	52.1	14594	12352	18.2	6.97	8.88	21.5
		W2N1	43	43	0	148	144	2.8	6126	5128	19.5	11738	14289	17.9	6.27	8.58	26.9
		W3N1	43	42	2.4	151	144	4.9	8338	5414	54.0	14627	14343	2.0	7.1	9.03	21.4
		CK	43	43	0	148	144	2.8	5996	4618	29.8	11544	12183	5.2	6.09	8.19	25.6
		Average			1.4			3.5			24.7			12.9			21.5

Note: Sim. and Obs. represent the simulated and observed values.
